# Structural, Thermal, and Electrical Properties of Poly(Ethylene Oxide)—Tetramethyl Succinonitrile Blend for Redox Mediators

**DOI:** 10.3390/polym14183728

**Published:** 2022-09-07

**Authors:** Ravindra Kumar Gupta, Hamid Shaikh, Ahamad Imran, Idriss Bedja, Abdullah Saleh Aldwayyan

**Affiliations:** 1 King Abdullah Institute for Nanotechnology, King Saud University, Riyadh 11451, Saudi Arabia; 2 SABIC Polymer Research Center, College of Engineering, King Saud University, Riyadh 11421, Saudi Arabia; 3 Cornea Research Chair, Department of Optometry, College of Applied Medical Sciences, King Saud University, Riyadh 11433, Saudi Arabia; 4 Department of Physics and Astronomy, College of Science, King Saud University, Riyadh 11451, Saudi Arabia; 5 K.A. CARE Energy Research and Innovation Center, King Saud University, Riyadh 11451, Saudi Arabia

**Keywords:** dye-sensitized solar cell, tetramethyl succinonitrile, polymer electrolyte, blend, redox mediator

## Abstract

An all-solid–state dye-sensitized solar cell is one of the non-fossil fuel-based electrochemical devices for electricity generation in a high-temperature region. This device utilizes a redox mediator, which is a fast ion-conducting solid polymer electrolyte (SPE). The SPE makes the device economical, thinner, and safer in high-temperature regions. The SPE generally has a form of matrix−plasticizer−redox salts. Succinonitrile (SN) is generally employed as a plasticizer for reducing the crystallinity of poly(ethylene oxide), abbreviated as PEO, a common polymeric matrix. In the present paper, the structural and thermal properties of tetramethyl succinonitrile (TMSN) were compared with SN for its application as a solid plasticizer. TMSN and SN both are plastic crystals. TMSN has four methyl groups by replacing the hydrogen of the SN, resulting in higher molecular weight, solid–solid phase transition temperature, and melting temperature. We thoroughly studied the structural, thermal, and electrical properties of the [(1−*x*)PEO: *x*TMSN] blend for utilizing it as a matrix, where *x* = 0–0.25 in mole fraction. The FT-IR spectra and XRD patterns of the blends exhibited PEO-alike up to *x* = 0.15 mole and TMSN-alike for *x* > 0.15 mole. Differential scanning calorimetry revealed formation of a eutectic phase from *x* = 0.1 mole and phase separation from *x* = 0.15 mole. The blends with *x* = 0.1–0.15 mole had a low value of PEO crystallinity. Thermogravimetric analysis showed thermal stability of the blends up to 75 °C. The blends exhibited electrical conductivity, σ_25°C_ more than 10^−9^ S cm^−1^, and Arrhenius behavior (activation energy, ~0.8 eV) in a temperature region, 25–50 °C.

## 1. Introduction

A redox mediator is an integral part of the dye-sensitized solar cell (DSSC). The redox mediator, an I^−^/I_3_^−^ redox-couple-based electrolyte, regenerates dye via oxidation of iodide at the working electrode. The iodide is regenerated at the counter electrode. The electrolyte also inhibits back electron transfer reactions. In particular, the energy level alignment between the redox level and the ground state of the dye is crucial for charge transfer and dye regeneration. For a review on DSSC, see references [1,2,3,4,5]. An electrolyte can be in a form of liquid, gel, or solid. In the liquid state, an electrolyte has a form of MI−I_2_−solvent, where M is an alkali metal or organic cation. The solvent can be a polar aprotic liquid, an ionic liquid, or both. The liquid nature offers easy preparation of the electrolyte, a high electrical conductivity (σ_25°C_ ~ 10^−2^ S cm^−1^), a high triiodide apparent diffusion coefficient (~10^−6^ cm^2^ s^−1^), and good interfacial contacts with electrodes [1,2,3,4,5]. This has resulted in solar cell conversion efficiency (η) as high as 11.9%@100 mW cm^−2^ [6]. The Co^2+^/Co^3+^-based liquid electrolytes exhibited better cell performance, such as 13% for SM315 dye [7], 13.6% for ZL003 dye [8], and ~14.3% for ADEKA-1+LEG4 dyes [9]. The Cu^+^/Cu^2+^ redox mediators had η ~12.7% for R7+Y123 dyes and 13.1% for Y123+XY1 dyes [10,11]. The liquid nature of the electrolyte, however, enforces hermetic sealing in the DSSC. This is for sustaining the high-pressure build-up because of a large diurnal temperature fluctuation in the Gulf countries, having a long summer with a temperature range of 40–50 °C. This also makes device manufacturing difficult. A gel electrolyte absorbs a large amount of liquid electrolyte in an organic or inorganic framework. This, therefore, possesses the electrical and interfacial properties similar to a liquid electrolyte, and shortcomings as well. A solid electrolyte particularly in a polymeric form is currently of great interest in research work. This eliminates the shortcomings of liquid electrolyte-based DSSC and makes the device lighter, thinner, and safer in high-temperature regions. For a review on electrolytes, see references [12,13,14,15,16,17,18,19,20,21,22,23,24].

A solid polymer electrolyte (SPE) in the form of PEO−MI−I_2_ is generally used as a solid redox mediator [12,13,14,15,16,17,18,19,20,21,22,23,24]. The PEO is an abbreviation of a high molecular weight poly(ethylene oxide), which is an excellent matrix. This is due to its properties such as dielectric constant (ε) between 5 and 8 at 25 °C and the Gutmann donor number of 22, resulting in ionic salt dissociation; segmental motion of polymeric chains; ion transportation through its ethereal oxygen; the optimal spacing between coordinating ethereal oxygens for solvation of the lithium ions; a self-standing film formation; ecologically and biologically benign; low material cost; and high thermal stability (~200 °C) [25,26,27,28]. The pure PEO-based electrolytes, however, exhibit poor σ_25°C_-values (≤10^−5^ S cm^−1^), and thereby, η < 1% at 1 sun. This was probably due to a higher PEO crystallinity (χ) hindering the ion transport. For decreasing the χ-value to nearly zero, thereby increasing the σ_25°C_-value more than 10^−4^ S cm^−1^, the PEO was blended with a plasticizer [12,13,14,15,16,17,18,19,20]. This was a low molecular weight polymer [29,30,31], ionic liquid [14,32], biopolymer [17], nanoparticle [30,33,34,35,36], and plastic crystal [37,38,39,40].

Plastic crystals are made of weakly interacting molecules, forming a globular shape due to the orientational or conformational degree of freedom [41]. That is, crystals are either symmetrical around their center or give a sphere by rotation around an axis. The plastic crystal phase exists generally in the cubic form between a solid–solid phase transition temperature (*T*_PC_) and melting temperature (*T*_m_). Plastic crystals portray a strong diffuse intensity along with the sharp Bragg peaks in the XRD patterns. These also exhibit melting entropy less than 17 J K^−1^ mol^−1^, a continuous rise in the value of the dielectric constant on lowering of the temperature, and a wax-like nature. Succinonitrile (SN; N≡C−CH_2_−CH_2_−C≡N) is a low molecular weight (*M*_W_ = 80.09 g mol^−1^) plastic crystal, which possesses a body-centered cubic structure. The plastic crystal phase exists between −38 °C (*T*_PC_) and 58 °C (*T*_m_). This has two molecules in the unit cell in which the diagonal of the cube possesses the central C−C bond, and the center of the face keeps the N atom [42]. This structure permits the molecular jump. This also has two isomers, gauche and trans. The latter acts as an impurity for creating mono-vacancy in the lattice, resulting in molecular diffusivity. A solid electrolyte, SN−XY, was used for solid–state battery application, where the notations X and Y stand for a cation and an anion of an ionic salt, respectively [43,44]. The succinonitrile showed the solid solvent plasticizing property because of its higher values of molar enthalpy (139.7 kJ mol^−1^), donor number (14 kcal mol^−1^), and ε (55 at 25 °C and 63 at 58 °C) [43,44,45]. This also helped in cation transport through its negative partial-charged nitrile group [44]. These properties of the SN forced the blending of PEO and SN in an equal weight proportion [37]. The (PEO−SN) blend had better properties such as a higher σ_25__°C_-value, a lower χ-value, and higher thermal stability along with other beneficial properties of the constituents. Additionally, the (PEO−SN) blend ensued solid redox mediators with better properties, such as σ_25°C_ ≈ 3–7 × 10^−4^ S cm^−1^, transparency in visible and IR regions >95%, χ ~ 0%, thermal stability ~125 °C, and η ≈ 2–3.7% @100 mW cm^−2^ with N719 dye [37,38,39,40,46].

Recently, Gupta et al. [47] synthesized [(1−*x*)PEO_8_: *x*TMSN]−LiI−I_2_ solid redox mediators, where *x* (=0–0.25 mole) is the composition. The abbreviation, TMSN corresponds to a colorless tetramethyl succinonitrile, which belongs to the plastic crystal family too [41]. As the name implies, the TMSN has four methyl groups by replacing hydrogen atoms of the succinonitrile with a structure, N≡C−(CH_3_)_2_C−C(CH_3_)_2_−C≡N. This has higher values of *M*_W_ (136.2 g mol^−1^) and *T*_m_ (~171 °C) than those of the succinonitrile [45,48]. The (0.85PEO: 0.15TMSN) blend-based redox mediator achieved σ_25°C_ of 1 × 10^−4^ S cm^−1^ and pseudo-activation energy (*B*) of ≈ 0.083 eV. As indicated by the results of the XRD, differential scanning calorimetry (DSC), and FT-IR spectroscopy, this was due to a decrease in the χ-value, which was associated with a large increase in the relative area of ω_CH2_ mode. This redox mediator attained η of ~3.5%@100 mW cm^−2^, nearly seven times higher than that of the PEO_8_−LiI−I_2_ (η ~ 0.5%@100 mW cm^−2^). These results motivated us to compare the structural and thermal properties of TMSN with SN and study the effect of TMSN on the structural, thermal, and electrical properties of the [(1−*x*)PEO: *x*TMSN] blends, where *x* = 0–0.25 mole. These were carried out using FT-IR spectroscopy, XRD, DSC, thermogravimetric analysis (TGA), and impedance spectroscopy. We also included structural, thermal, and electrical properties of the SPE, [0.85 PEO_8_: 0.15 TMSM]−LiI−I_2_ for direct comparison [47].

## 2. Materials and Methods

### 2.1. Synthesis

The chemicals PEO (*M*_W_ ≈ 1 × 10^6^ g mol^−1^) and TMSN were procured from Sigma Aldrich, Inc., USA, and TCI, Japan, respectively, and used without purification. The conventional solution cast method was employed for obtaining a self-standing film of the PEO−TMSN blend, which had the steps of (a) a rigorous stirring of the PEO and TMSN in 20-mL acetonitrile at 65 °C for 48 h, (b) casting of the homogeneous solution on a Teflon Petri dish, (c) drying in a nitrogen gas atmosphere (2-weeks), and (d) drying in a vacuum (1 day). For detail on blend synthesis, please see reference [37].

### 2.2. Characterizations

*FT-IR spectroscopy*: A small amount of SN, TMSN, or PEO was homogeneously mixed with dried potassium bromide powder using an Agate pestle and mortar, followed by pelletizing using a die under 2 tons cm^−2^ of pressure. A thin film of the blend on the potassium bromide pellet was prepared following the method of Gupta et al. [37]. We collected the absorbance spectrum of the blend in a range of 400−4000 cm^−1^ (resolution, 1 cm^−1^) and analyzed using the EZ-OMNIC software (Thermo Scientific Inc., Waltham, MA, USA). We employed a Perkin Elmer, Spectrum 100, FT-IR spectrometer (Waltham, MA, USA) for the measurement.

*XRD*: A circular hole of the sample holder was filled in by the SN, TMSN, PEO, or blend film. The XRD pattern was collected using a Bruker, D2 Phaser, X-ray diffractometer (Karlsruhe, Germany), having the specifications: CuKα radiation, 1.54184 Å; range, 10–60°; and step, 0.06°.

*DSC*: A few milligrams of the blend was sealed in an aluminum pan. We employed a Shimadzu, DSC-60A, DSC unit (Kyoto, Japan) for the heat flow measurement of the blend. The heating rate was 10 °C min^−^^1^. This was performed under the purging of nitrogen gas.

*TGA*: We measured the weight loss of the blend in the temperature range of 25–600 °C under the purging of nitrogen gas. This was performed using a Shimadzu, DTG-60H, TGA unit (Kyoto, Japan) with a heating rate of 10 °C min^−1^_._

*Electrical conductivity*: Impedance spectroscopy was utilized for the σ measurement of the film, having a thickness of *l* and area of *A*. This was carried out using a Palmsens4 impedance analyzer (Houten, the Netherlands) with the experimental conditions, such as stainless steel plates as a blocking electrode, 20 mV AC voltage, and a frequency range of 100 kHz to 1 Hz. The Nyquist curve yielded values of bulk resistance and thereby σ [38].

## 3. Results and Discussion

### 3.1. Comparison of TMSN with SN

**Figure 1:**
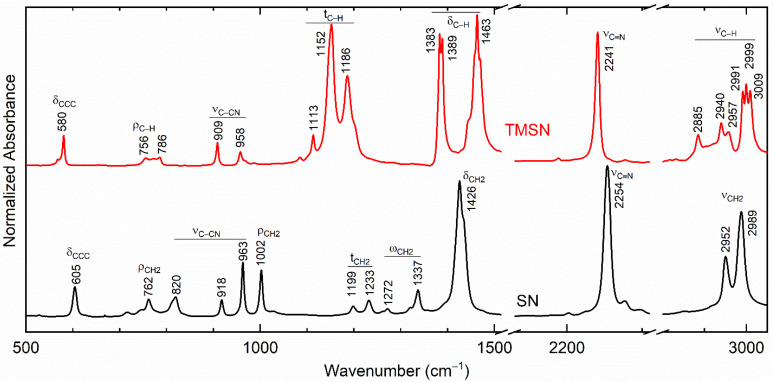
FT-IR spectra of SN and TMSN.

Figure 1 shows the FT-IR spectrum of SN, which has sharp vibrational peaks because of the crystalline nature of molecules. The peak values are similar to that reported earlier by Fengler and Ruoff [49] and, therefore, are assigned accordingly. The vibrational modes were denoted by ν, δ, ω, t, and ρ, which correspond to stretching, bending, wagging, twisting, and rocking modes, respectively. Figure 1 also portrayed the spectrum of TMSN. The TMSN possesses the CH_3_ groups by replacing H of the succinonitrile. This is, therefore, similar to that of succinonitrile with an influence of the methyl group, resulting in a redshift in several modes and the crystal field splitting phenomenon because of the doubling of the methyl group [50].

**Figure 2:**
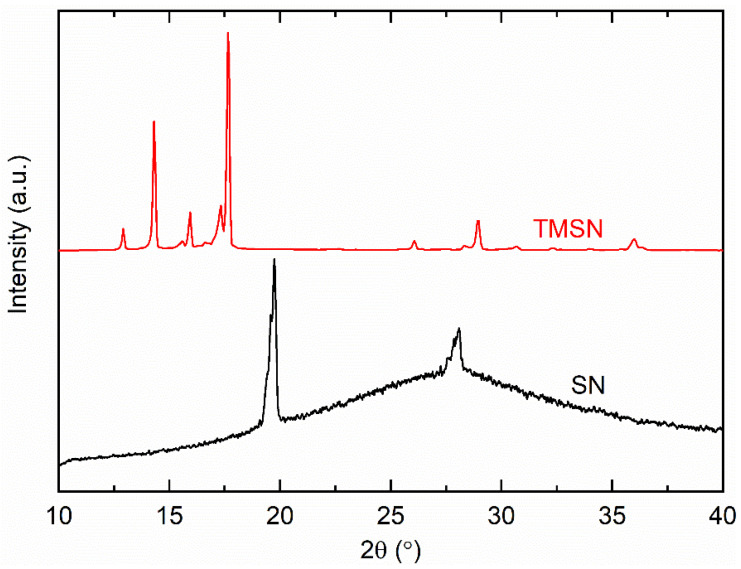
XRD patterns of SN and TMSN.

Figure 2 exhibits XRD patterns of SN and TMSN. As mentioned earlier, SN has a bcc structure [42]. This, therefore, portrayed a strong reflection peak at ~20° and a medium peak at ~28° as well as a defuse peak [37,51]. The replacement of H of the succinonitrile by CH_3_ resulted in TMSN with an XRD pattern having the reflection peaks at ~12.9, 14.3, 15.9, 17.6, and 29°. A thorough Rietveld analysis is, however, required to ascertain its bcc nature.

**Figure 3:**
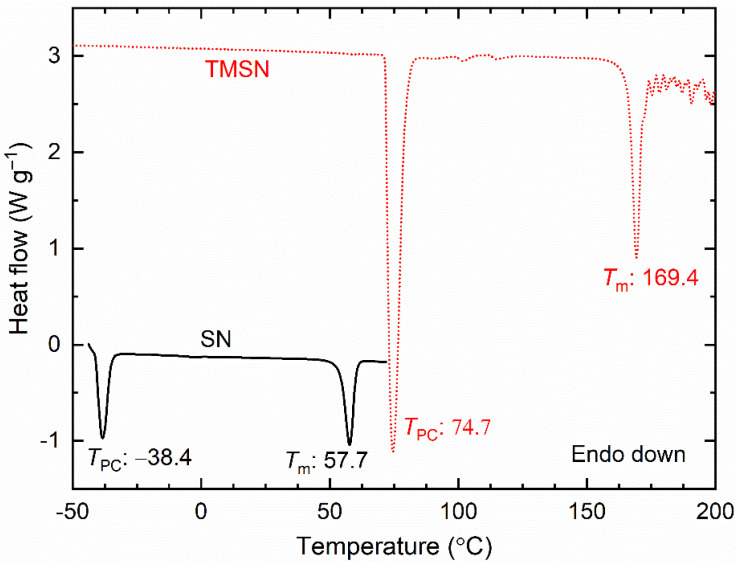
DSC curves of SN and TMSN.

Figure 3 shows the DSC curves of SN and TMSN. The low molecular weight plastic crystal SN exhibited values of *T*_PC_ and *T*_m_ at −38 and ~58 °C, respectively, similar to those reported earlier [43,44,51]. The replacement of H of the succinonitrile by CH_3_ led to TMSN with a higher molecular weight. This resulted in higher values of *T*_PC_ (~75 °C) and *T*_m_ (~169 °C), and was noted to be similar to those reported earlier [45,48].

### 3.2. Blend’s Characterizations

#### 3.2.1. Structural properties

**Figure 4:**
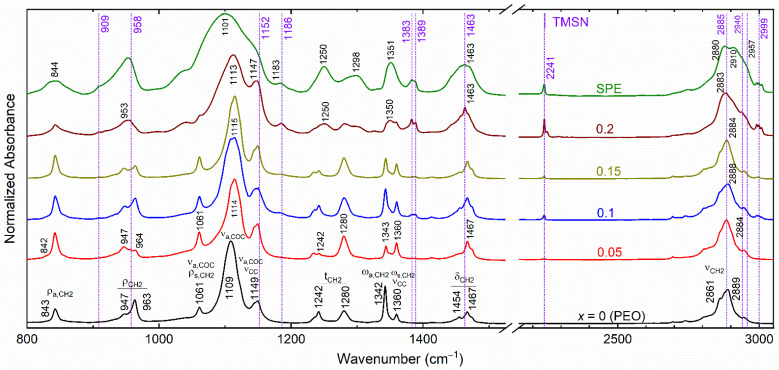
FT-IR spectra of the [(1−*x*)PEO: *x*TMSN] blends with *x* = 0–0.2 mole and SPE. The dotted vertical lines correspond to peaks of TMSN.

Figure 4 exhibits FT-IR spectra of the [(1−*x*)PEO: *x*TMSN] blends with *x* = 0–0.2 mole. The observed modes of TMSN are represented by dotted vertical lines for direct comparison. We included the FT-IR spectrum of the SPE for comparison too. The spectrum of the PEO (*x* = 0 mole) is similar to that reported earlier by Yoshihara et al. [52]. The peaks are, therefore, assigned accordingly using the conventional notations: ν (stretching), δ (bending), ω (wagging), t (twisting), ρ (rocking), s (symmetric), and a (asymmetric). The addition of TMSN (*x* = 0.05–0.15 mole) unaffected the position of several modes of PEO in the figure-print and ν_C__≡__N_ regions significantly except for the ν_a,COC_ mode at ~1114 cm^−1^. The addition, however, changed the position of the ν_a,CH2_ mode of PEO from 2889 to 2884 cm^−1^ for the blends with *x* = 0.05 and 0.15 mole. This indicates an increase in the C−H bond of the PEO due to the PEO-TMSN interaction [50]. The addition of 0.2 mole, however, significantly changed the position of several modes. For example, the ρ_CH2_ modes of PEO at 947 and 963 cm^−1^ unified at 953 cm^−1^; the ν_a,COC_ mode of PEO shifted to 1113 cm^−1^; the t_CH2_ mode of PEO at 1242 cm^−1^ shifted to 1250 cm^−1^; the ω_CH2_ modes of PEO at 1342 and 1360 cm^−1^ unified at 1350 cm^−1^; the δ_CH2_ modes of PEO at 1454 and 1467 cm^−1^ unified at 1463 cm^−1^; and the ν_a,CH2_ mode of PEO shifted to 2883 cm^−1^. This also had broadened peaks for the PEO-based modes and quite prominent TMSN-based modes. These demonstrate a significant change in the PEO conformation after *x* = 0.15 mole [53], as observed by the XRD study, which is discussed later.

The SPE, having the blend (*x* = 0.15 mole) and redox salts, observed a significant change in the PEO structure relative to that of the pure blend. For example, the ρ_CH2_ modes at 947 and 964 cm^−1^ unified at 953 cm^−1^; the ν_a,COC_ mode at 1115 cm^−1^ shifted to 1101 cm^−1^ along with the merging of the shoulder peaks at 1061 and 1149 cm^−1^; the t_a,CH2_ mode at 1242 cm^−1^ shifted to 1250 cm^−1^; the t_s,CH2_ mode at 1280 cm^−1^ shifted to 1298 cm^−1^; the ω_CH2_ modes at 1342 and 1360 cm^−1^ unified at 1351 cm^−1^; the δ_CH2_ modes at 1454 and 1467 cm^−1^ unified at 1463 cm^−1^; and the ν_a,CH2_ mode shifted to 2885 cm^−1^. The PEO bands were noted as quite broad. These observations were similar to those of the bend with *x* = 0.2 mole, revealing the plasticizing effect of the salt, i.e., the existence of amorphous regions for ion transport [37,38,39,53]. These changes are due to the salt–PEO–TMSN interactions [37,38,39].

**Figure 5:**
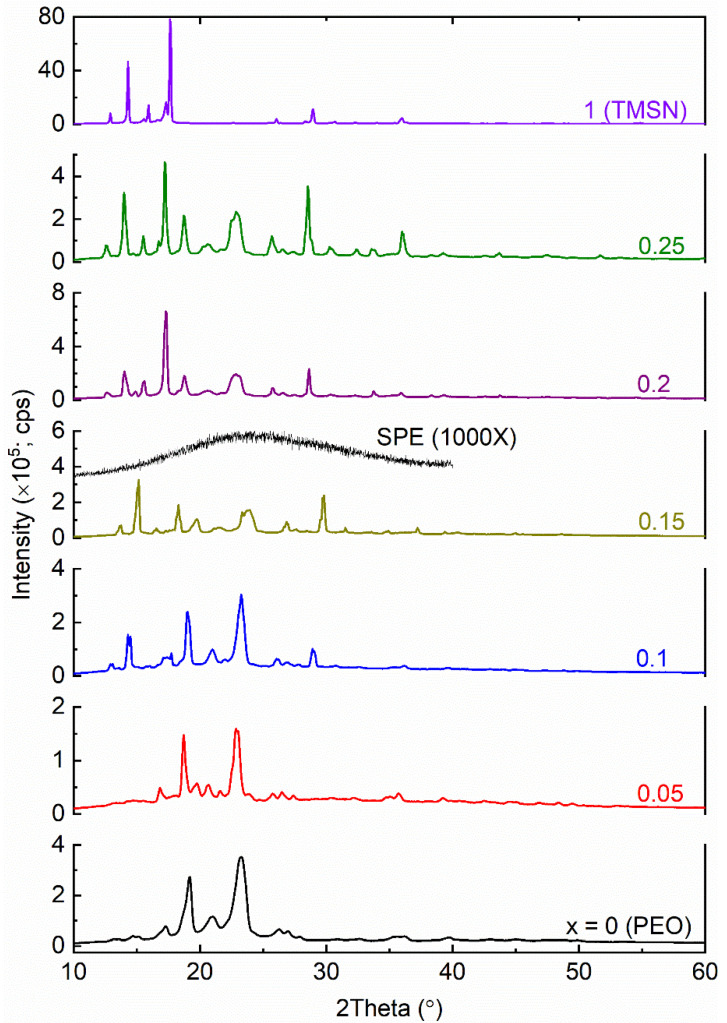
XRD patterns of the [(1−*x*)PEO: *x*TMSN] blends (*x* = 0–0.25, and 1 mole) and SPE.

**Figure 6:**
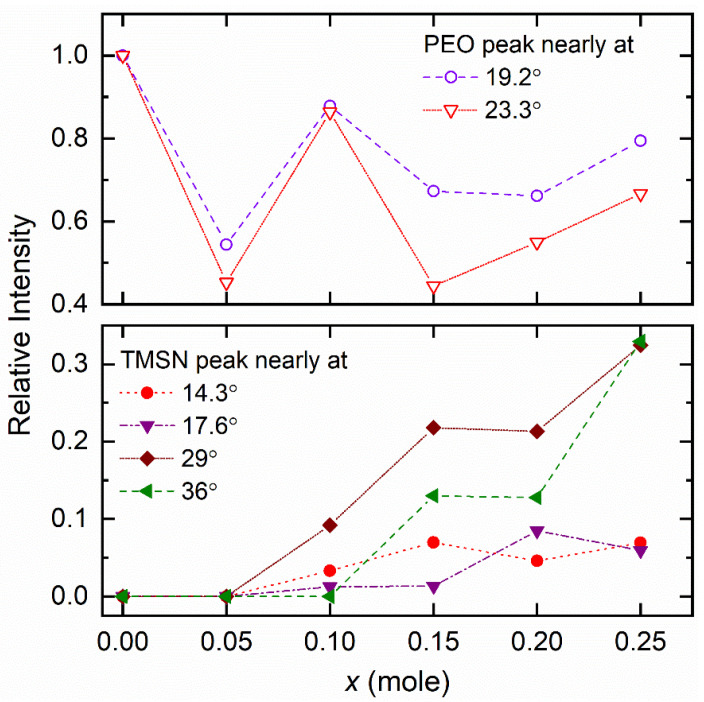
Relative intensity of peaks of PEO and TMSN in the [(1−*x*)PEO: *x*TMSN] blends, where *x* = 0–0.25 mole.

Figure 5 shows XRD patterns of the [(1−*x*)PEO: *x*TMSN] blends, where *x* = 0–0.25, and 1 mole. Having long linear −CH_2_−CH_2_−O− chains, the high molecular PEO (*x* = 0 mole) is semi-crystalline and exhibits strong reflection peaks at 2θ of 19.2° and 23.3° [37]. The blending of the PEO with TMSN significantly changed the position and intensity of the reflection peaks of both the PEO and TMSN. The change is shown in Table 1 and Figure 6 for direct comparison. The blend with *x* = 0.05 mole observed a decrease in the 2θ-value and intensity of PEO, revealing an increase in spacing between polymeric chains and thereby a decrease in PEO crystallinity [40,54]. A further increase in *x*-value to 0.1 mole led to a slight increase in 2θ-value, although it still had a low intensity compared to the PEO, indicating restructuring of the PEO chains to accommodate TMSN molecules. The blend with *x* = 0.15 mole portrayed a peak shift to 18.3° with a lower intensity, revealing a further decrease in PEO crystallinity. This had TMSN peaks at higher 2θ-values compared to those of the pure TMSN and the TMSN intensities higher than those of the PEO. The blends with *x* = 0.2 and 0.25 mole showed PEO peaks at the 2θ of ~18.8° and ~22.9° with intensities higher than those of the blend with *x* = 0.15 mole and lower than those of the pure PEO; however, TMSN peaks became prominent and shifted close to those of the pure TMSN. These indicated recrystallizations of PEO chains and TMSN molecules in the blends, respectively, i.e., the phase separation phenomenon, resulting in an increase in crystallinity of PEO and TMSN for *x* > 0.15 mole as observed by the DSC study and discussed later. One can also observe that the blends with *x* ≤ 0.15 mole are PEO-alike, and blends with *x* > 0.15 mole are TMSN-alike. The blends with *x* = 0.1–0.25 mole also had an additional peak at 2θ range of 25.7–26.8° apart from a shift in 2θ-values of PEO and TMSN peaks, indicating a eutectic phase formation [44]. Figure 5 also shows the XRD pattern of the SPE without the reflection peaks of the PEO, TMSN, LiI, and I_2_. The absence of peaks indicated the formation of the amorphous phase in the SPE, which is due to the salt–PEO–TMSN interactions [37,38,39]. The DSC revealed a similar result, which is discussed below.

#### 3.2.2. Thermal properties

**Figure 7:**
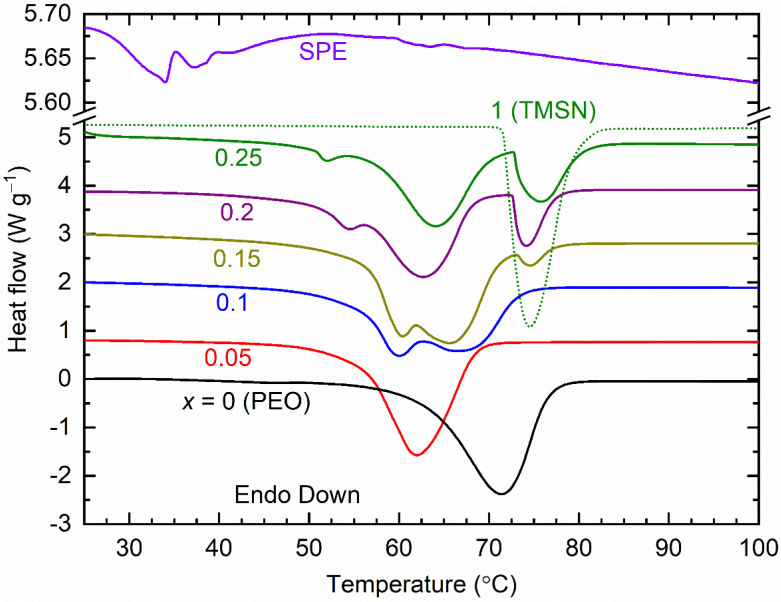
DSC curves of the [(1−*x*)PEO: *x*TMSN] blends (*x* = 0–0.25, and 1 mole) and SPE.

**Figure 8:**
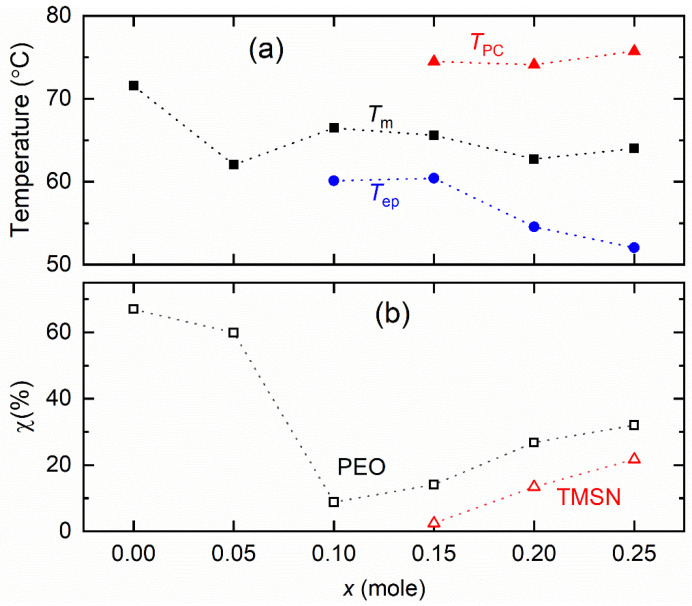
(**a**) Values of *T*_m_, *T*_ep_, and *T*_PC_ of the [(1−*x*)PEO: *x*TMSN] blends, where *x* = 0–0.25 mole. (**b**) Crystallinity (χ) of PEO and TMSN for the blends. For details, see the text.

Figure 7 shows the DSC curves of the [(1−*x*)PEO: *x*TMSN] blend (*x* = 0–0.25, and 1 mole) and SPE. Being a semi-crystalline polymer, the PEO (*x* = 0 mole) exhibited a strong endothermic peak at 71.5 °C, which is the melting point (*T*_m_) of the PEO [37]. The introduction of a small amount of TMSN in PEO (*x* = 0.05 mole) led to a decrease in the *T*_m_-value to 62 °C of the blend. A further increase in *x* (= 0.1–0.25 mole) resulted in a secondary endothermic peak (*T*_ep_) because of the eutectic phase formation [44] along with the *T*_m_-peak of the blend. The blends with *x* = 0.15–0.25 mole were accompanied by a third endothermic peak nearly at 74.5 °C, falling to the domain of the *T*_PC_-peak of the TMSN. We constructed a phase diagram as shown in Figure 8a. This depicted the *T*_m_-value range between 62 and 66.6 °C with the *T*_ep_-peak at ~60 °C for blends with *x* = 0.1 and 0.15 mole, 54.6 °C for *x* = 0.2 mole, and 52 °C for *x* = 0.25 mole, revealing the eutectic phase formation as observed earlier by the XRD study. The existence of the *T*_PC_-peak for blends with *x* = 0.15–0.25 mole indicated the phase separation phenomenon, in which the phase separation is the lowest for the blend with *x* = 0.15 mole. We estimated the crystallinity (χ) of PEO and TMSN for the blends using an expression, χ = 100 Δ*H*/*H*_pure_. This has notations, *ΔH* for the heat enthalpy of the blend at *T*_m_ or *T*_PC_, and *H*_pure_ for the heat enthalpy of fully crystalline PEO (193 J g^−1^ [37]) or TMSN (134.8 J g^−1^). Figure 8b shows crystallinity of PEO and TMSN for the blends with *x* = 0–0.25 mole. The χ-value for the PEO decreased sharply up to *x* = 0.1 mole and then increased relatively slowly and linearly for higher values of *x*. The χ-value for the TMSN observed a similar trend for the blends with *x* = 0.15–0.25 mole. This suggested that the composition, *x* = 0.1–0.15 mole, can be utilized as a host matrix for the synthesis of a solid polymer electrolyte. Figure 7 also shows the DSC curve of the SPE for comparison. This portrayed a significant reduction in the heat enthalpy area and existence of the bi-endothermic peaks in the temperature region, 32–40 °C relative to the matrix (*x* = 0.15 mole). This indicated the eutectic phase formation with a small PEO crystallinity (1.7%) [47]. This curve had no *T*_PC_ peak either, revealing the salt–PEO–TMSN interactions [37,38,39].

**Figure 9:**
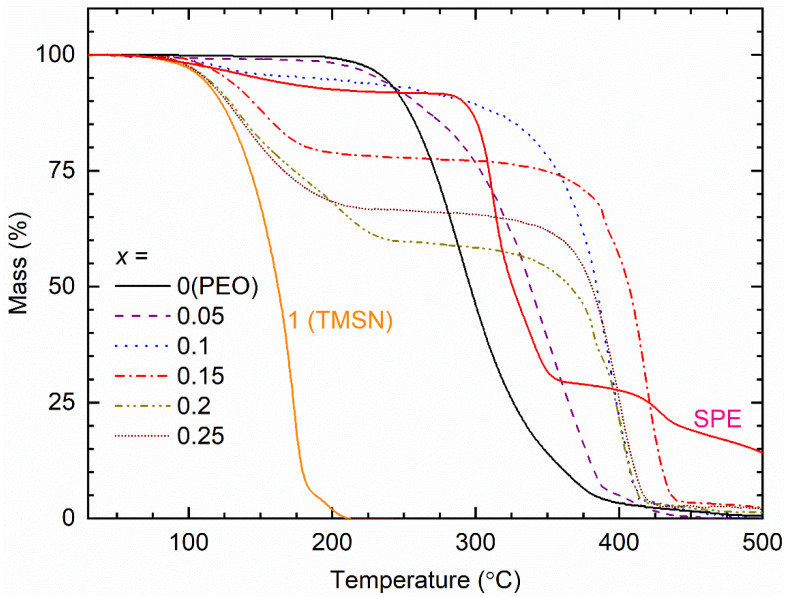
TGA curves of the [(1−*x*)PEO: *x*TMSN] blends (*x* = 0–0.25, and 1 mole) and SPE.

Figure 9 shows TGA curves of the [(1−*x*)PEO: *x*TMSN] blends (*x* = 0–0.25 mole) and pure TMSN (*x* = 1 mole) for comparison. The PEO portrayed a flattened region up to ~200 °C and a huge drop (~95%) from 200 to 400 °C. The initial flattened region is the thermal stability of the polymer, and the drop is due to the degradation/burning out of the polymer [37,38,39,55]. The degradation products comprised formaldehyde, ethanol, low molecular weight poly(ethylene oxide), carbon dioxide, water, and nearly 20 saturated and unsaturated compounds in the range of one to seven carbons [55,56]. The addition of a small amount of TMSN (*x* = 0.05 mole) unaltered the thermal stability of the blend significantly; instead, it slowed down the rate of degradation/burning out of the polymer because of the composite phenomenon [57,58]. The blends with *x* = 0.1–0.25 mole, however, showed the following regions, (i) a plateau up to ~75 °C, (ii) a small drop, (iii) a near plateau region, and (iv) a large drop. These steps were dependent on the value of *x*. As visualized, the initial drop was due to the TMSN degradation/sublimation, which is the lowest for the blend with *x* = 0.1 mole and the highest for the blend with *x* = 0.2 mole [37]. As mentioned earlier, the final drop was due to the thermal degradation of the PEO. It seems that the eutectic phase formation and phase separation played crucial roles in controlling the thermal property of the blends. The (PEO−TMSN) blend showed thermal stability up to ~75 °C, which is lower than that of the (PEO−SN) blend (~125 °C [37]). This is due to the CH_3_ group that weakened the associated C−C bonding of the TMSN [55]. Figure 9 also shows the TGA curve of the SPE, which consisted of the matrix and redox salts. It exhibited the usual pattern, (i)−(iv). The initial drop is, however, less than that of the matrix because of the redox salts [37,58]. The SPE had an initial plateau region similar to that of the matrix, making the device operational up to 75 °C.

#### 3.2.3. Electrical Transport Properties

**Figure 10:**
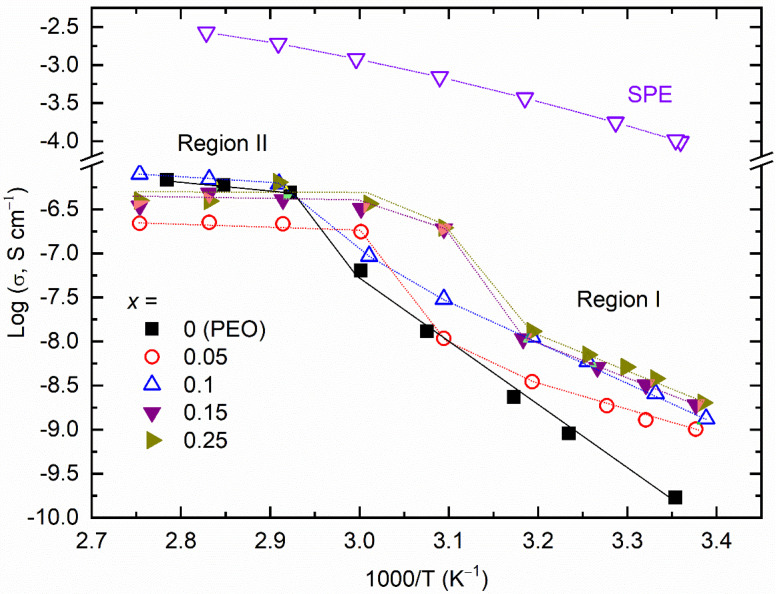
Log σ − *T*^−1^ plots for the [(1−*x*)PEO: *x*TMSN] blends (*x* = 0–0.25 mole) and SPE.

Figure 10 shows log σ − *T*^−1^ plots for the [(1−*x*)PEO: *x*TMSN] blends, where *x* = 0–0.25 mole. This had temperature regions I and II before and after the melting temperature of the blends, respectively. The PEO (*x* = 0 mole) exhibited a low value of σ_25__°__C_ (1.7 × 10^−10^ S cm^−1^), because of the segmental motion of long polymeric chains [25,26,27,28,37]. An increase in temperature increased the σ-value linearly in both regions. Region II had a σ-value higher than that in region I because region II is the amorphous region [25,26,27,28,37]. The linear log σ − *T*^−^^1^ curve is expressed by the Arrhenius equation, σ = σ_o_ exp[−*E*_a_/k_B_*T*] [25,26,27,28,37]. This has notations, σ_o_ for a pre-exponential factor and k_B_ for the Boltzmann constant. The slope of the linear curve yielded the activation energy (*E*_a_) with a value of 1.44 eV in region I and 0.21 eV in region II. The blending of PEO with TMSN did not change the log σ − *T*^−^^1^ pattern. However, it improved the electrical transport properties of the blends. For examples, the σ_25__°__C_-value increased to 1 × 10^−9^ S cm^−1^ for *x* = 0.05 mole, 1.3 × 10^−9^ S cm^−1^ for *x* = 0.1 mole, 1.9 × 10^−9^ S cm^−1^ for *x* = 0.15 mole, and 2 × 10^−9^ S cm^−1^ for *x* = 0.25 mole. The *E*_a_-value reduced to 0.73 eV (region I) and 0.07 eV (region II) for *x* = 0.05 mole, 0.84 eV (region I) and 0.14 eV (region II) for *x* = 0.1 mole, 0.76 eV (region I) and 0.04 eV (region II) for *x* = 0.15 mole, and 0.82 eV (region I) and 0.01 eV (region II) for *x* = 0.25 mole. This trend is similar to that observed earlier for the PEO−SN blend [37]. As suggested by the structural and thermal studies, the improvement is due to the formation of the eutectic phase with a low PEO crystallinity. This is controlled by the phase separation phenomenon.

Figure 10 also exhibited the log σ − *T*^−1^ pattern of the [0.85PEO: 0.15TMSN]−LiI−I_2_ SPE. The SPE possessed σ_25__°__C_ of 1 × 10^−4^ S cm^−1^, nearly five orders higher than that of the matrix. The log σ − *T*^−1^ pattern was downward. These behaviors of the SPE are similar to those observed earlier for the (PEO−SN)−MI−I_2_ solid redox mediators, which exhibited σ_25__°__C_ of 3 × 10^−4^ S cm^−1^ for M = Li and 7 × 10^−4^ S cm^−1^ for M = K [38,39]. Recently, Gupta et al. [59] reported σ_25__°__C_ ~ 7 × 10^−4^ S cm^−1^ with the downward log σ − *T*^−^^1^ pattern for the solid Co^2+^/Co^3+^ redox mediator, (PEO−SN)−LiTFSI−Co(bpy)_3_(TFSI)_2_−Co(bpy)_3_(TFSI)_3_. The downward nature revealed the supremacy of the amorphous regions over the semi-random motion of short polymer chains for ion transport. This finding is similar to the FT-IR spectroscopy, XRD, and DSC results. The amorphous domains-dominated ion transport phenomenon follows the Vogel–Tamman–Fulcher empirical relation: σ = σ_o_*T*^−^^½^ exp[−*B*/ k_B_(*T* − *T*_o_)], where *T*_o_ is the temperature at which the free volume vanishes [25,26,27,28,38,39,59]. The linear curve of log σ*T*^½^ vs. (*T* − *T*_o_)^−1^ plot results in the pseudo-activation energy (*B*), which is 0.083 eV, similar to those observed for the (PEO−SN) blend-based solid redox mediators [38,39,59]. This value is less than 0.3 eV, a requirement for the device application [57].

## 4. Conclusions

Being members of plastic crystals, structural and thermal properties of TMSN and SN were compared. TMSN has four methyl groups by replacing the hydrogen atom of the SN. This replacement resulted in a higher molecular weight, thereby the higher *T*_m_ and *T*_PC_ values. The TMSN had five reflection peaks with lower 2θ-values instead of two peaks of the SN in the XRD pattern. This also led to a redshift in several vibrational modes and the crystal field splitting phenomenon. A partial replacement of PEO by TMSN with 0.1 to 0.15 mole was noted as useful in terms of the eutectic phase formation with a low PEO crystallinity, a low phase separation, thermal stability up to 75 °C, an order higher σ_25°C_-value, and nearly half of the activation energy of the PEO. A solid polymer electrolyte, utilizing [0.85PEO: 0.15TMSN] blend as a matrix and LiI−I_2_ as redox salts, exhibited σ_25°C_ of ~1 × 10^−4^ S cm^−1^ with a pseudo-activation energy of ~0.08 eV because of the formation of the amorphous phase.

## Figures and Tables

**Table 1:** The peak position of ingredients in the XRD patterns of the [(1−*x*)PEO: *x*TMSN] blends.

*x* (Mole)	PEO (°)		TMSN (°)					
0	19.2	23.3	-	-	-	-	-	-
0.05	18.7	22.8	-	-	-	-	-	-
0.1	19	23.3	13.1	14.3	17.5	21.0	28.9	-
0.15	18.3	23.9	13.8	15.2	16.5	19.8	29.8	37.2
0.2	18.8	22.9	12.6	14.0	15.5	17.3	28.6	35.9
0.25	18.7	22.8	12.5	14.0	15.5	17.2	28.5	36.0
1	-	-	12.9	14.3 s ^†^	15.9	17.6 vs ^†^	29.0	36.0

^†^ Notations, s and vs stand for strong and very strong intensity, respectively.

## Data Availability

Data are available upon request from the corresponding author.
